# Restrictive allogeneic blood transfusion strategy in patients with extremity bone sarcomas

**DOI:** 10.1097/MD.0000000000018482

**Published:** 2019-12-20

**Authors:** Jong Hoon Park, Seok Ha Hong, Woo Young Jang

**Affiliations:** Department of Orthopedic Surgery, Korea University Anam Hospital, Seoul, Republic of Korea.

**Keywords:** allogeneic blood transfusion, extremity bone sarcoma, restriction

## Abstract

Allogeneic blood transfusions (ABTs) are common in patients with cancer. The present study investigated the safety of a restrictive ABT strategy in patients with extremity sarcomas.

Patients who underwent operations for extremity bone sarcomas between May 2008 and November 2018 were retrospectively reviewed. Clinical outcomes based on hemoglobin concentrations, postoperative infections, and hospital stay were compared between 20 patients who received liberal ABT (control group) and 19 patients who received restrictive ABT (restrictive group). The rates of distant metastasis and death were compared between the groups.

The mean number of ABTs was 3.6 ± 3.8 units in the control group and 0.33 ± 0.74 units in the restrictive group (*P* < .001). Only 3 of 19 patients received transfusions (2 red cell packs each). The hemoglobin levels tended to fall during the first 3 postoperative days but seemed to stabilize within the first postoperative week in both groups. Postoperative surgical site infections only occurred in the patients who received ABTs regardless of the group. The rates of distant metastasis and death were higher in the control group than in the restrictive group (25.0% vs 15.7% and 10.0% vs 0%, respectively), but the differences were not significant.

A restrictive ABT strategy may be safely performed in patients with extremity bone sarcomas depending on the intraoperative status and specific characteristics of each patient.

## 1 Introduction

Allogeneic blood transfusion (ABT) is a routine medical procedure used to increase patient hemoglobin (Hb) levels.^[1]^ An accurate understanding of the clinical outcomes of perioperative ABT is essential in patients with extremity bone sarcomas (EBSs) because the potential need for perioperative ABTs is high due to anemia caused by preoperative chemotherapy and blood loss during extensive resections in such patients. Up to 40% of patients with cancer receive perioperative ABT.^[2]^

Perioperative ABT is considered a lifesaving procedure due to the resultant oxygen delivery and tissue perfusion improvements. However, ABT may also result in an altered immune response or transfusion-related acute lung injury or thrombosis. Therefore, the transfusions may adversely affect surgical outcomes such as postoperative infection, morbidity, and length of hospital stay.^[3]^ Furthermore, many previous reports have demonstrated that perioperative ABT negatively impacts postoperative cancer survival rates in patients with colorectal carcinomas^[4–6]^ as well as other cancer types.^[7–9]^ For these reasons, many studies have used a restrictive ABT strategy in such patients.^[10–12]^

In terms of the impact of ABT on patients with EBSs, several studies^[13–15]^ also reported that perioperative ABT was a poor prognosis predictor for distant metastasis and survival in patients with EBSs. However, little information is available in the literature regarding the impact of a restrictive ABT strategy on patients with EBSs. Hence, we sought to examine the efficacy, safety, and clinical results of a restrictive ABT strategy in patients with EBSs. We hypothesized that a restrictive ABT strategy would be safe and feasible in patients with EBSs.

## 2 Methods

### 2.1 Patients

Following approval by the Institutional Review Board of Korea University Hospital (2018AN0107), the medical records of patients who underwent operations for EBSs between May 2008 and November 2018 were retrospectively reviewed. We identified 70 patients who underwent EBS surgery between 2008 and 2018; 29 were excluded because of metastatic lesions, leaving the 39 patients included in this study. Prior to 2013, we routinely performed liberal ABTs in patients with EBSs; however, in 2013, patient blood management guidelines were revised to include restrictive transfusions. In this study, a restrictive transfusion strategy (ABTs were administered if Hb levels were <7.0 g/dL or from 7 to 10 g/dL in the presence of anemia symptoms such as tachycardia or dyspnea) was compared with a liberal strategy.

### 2.2 Patient blood management protocol

When preoperative Hb levels are <12 g/dL (women) or <13 g/dL (men), we consider the patient to have preoperative anemia, according to a previous report.^[16]^ In patients with preoperative anemia, preoperative supplemental intravenous iron (500 mg as ferric carboxymaltose) and recombinant human erythropoietin (rHuEPO, 4000 U) were administered. Intraoperative blood loss minimization strategies were also employed, including hypothermia prevention and induced hypotension; tranexamic acid, an antifibrinolytic agent, was also administered, according to the Association of Anaesthetists of Great Britain and Ireland guidelines. Postoperatively, ABT triggers were Hb levels <7 g/dL or Hb levels of 7 to 10 g/dL in the presence of anemia symptoms such as tachycardia or dyspnea.

### 2.3 Clinical outcomes analysis

The American Society of Anesthesiologists (ASA) score^[17]^ was determined to evaluate each patient's general morbidity. To investigate the clinical outcomes of the restrictive ABT strategy, Hb concentrations, postoperative surgical site infections, and hospital stay duration were compared between the 20 patients who received liberal ABTs (control group) and the 19 patients who received restrictive ABTs (restrictive group). The rates of postoperative distant metastasis and death were also compared between the groups. All continuous variables are reported as means ± standard deviations. The chi-squared test was used to compare categorical variables between the groups, and Student *t* tests were performed to compare the means of 2 independent variables. *P* values <.05 were considered significant.

## 3 Results

Demographic data are presented in Table 1. There were no significant between-group differences in sex distribution, age, tumor grade, or tumor size. Moreover, the mean preoperative hemodynamic value and ASA score showed no significant differences between the groups (Table 2). However, the mean number of ABTs were 3.6 ± 3.8 units in the control group and 0.33 ± 0.74 units in the restrictive group (*P* < .001). In the restrictive group, only 3 of 19 patients received transfusions, and each received 2 red blood cell packs. Hb levels tended to fall during the first 3 postoperative days but seemed to stabilize within the first postoperative week in both groups.

**Table 1:**
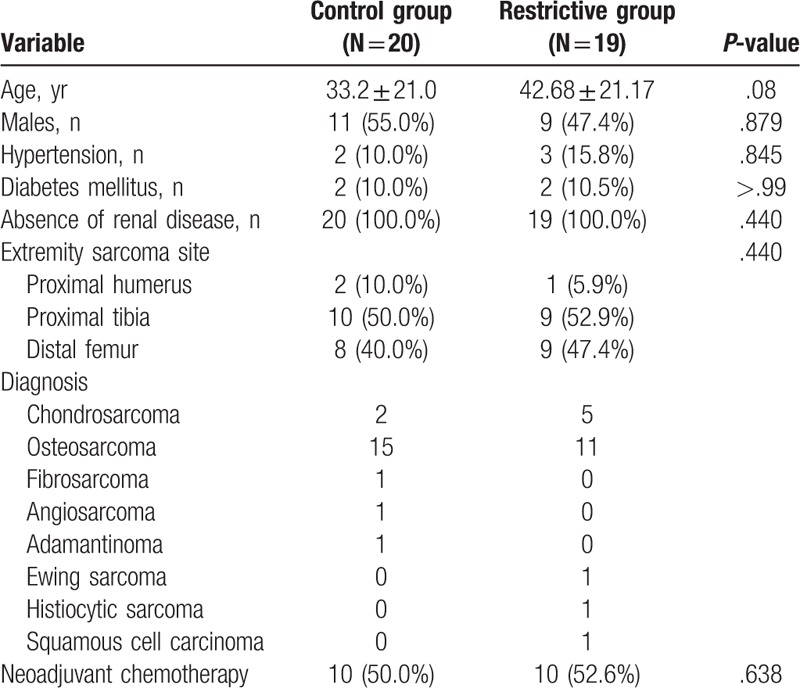
Baseline patient characteristics.

**Table 2:**
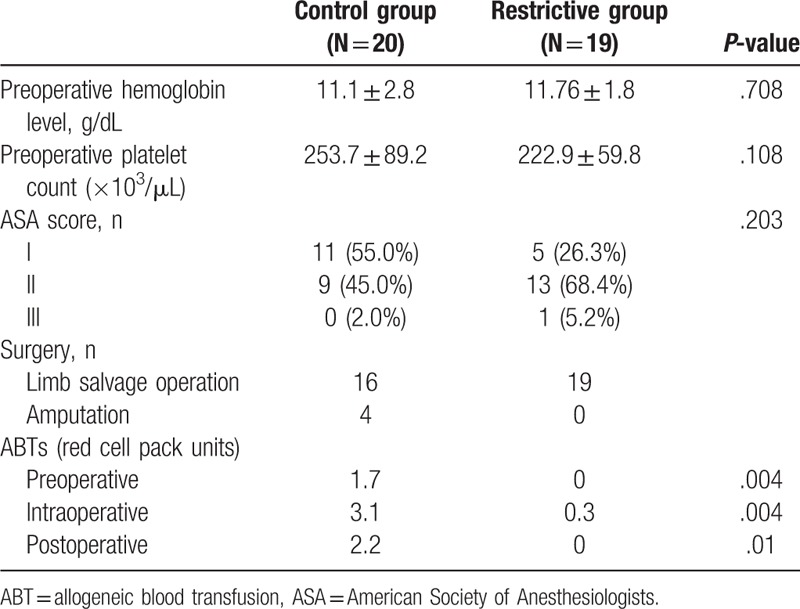
Hemodynamic variables and operation types in the liberal allogeneic blood transfusion (control group) and restrictive allogeneic blood transfusion (restrictive group) groups.

The postoperative clinical outcomes failed to show any significant differences between the 2 groups (Table 3). Postoperative surgical site infections occurred in 6 patients receiving ABTs: 4 patients in the control group and 2 patients in the restrictive group. The rates of distant metastasis and death were higher in the control group than in the restrictive group (18.2% vs 6.7% and 9.1% vs 0%, respectively), but the differences were not significant.

**Table 3:**
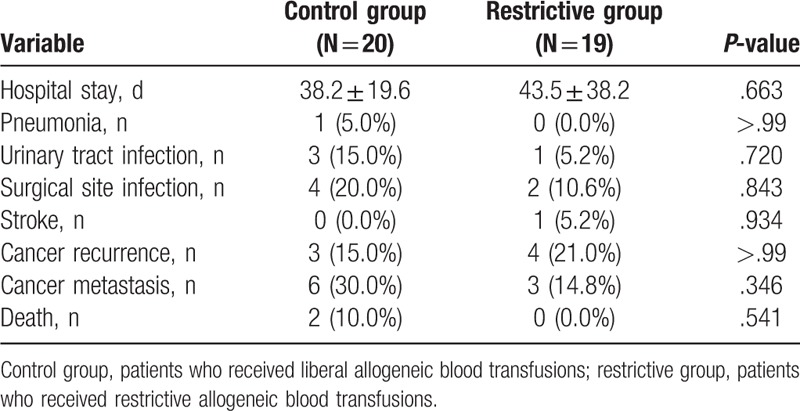
Postoperative clinical outcomes.

## 4 Case

**Figure 1:**
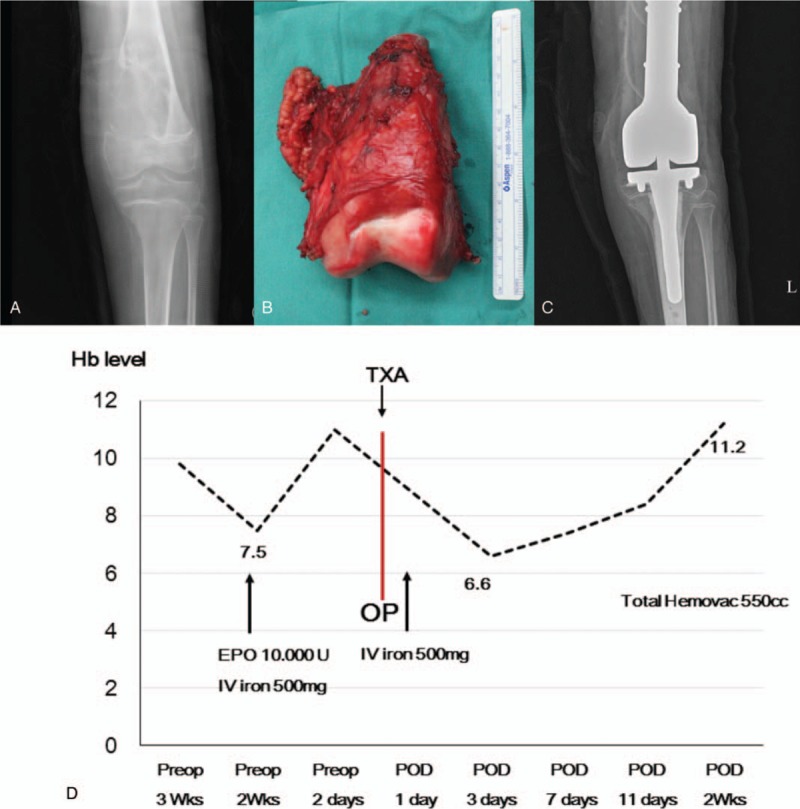
An 11-year-old boy with a left distal femur osteosarcoma underwent a wide-excision and limb-salvage operation with tumor prosthesis, using a restrictive allogeneic blood transfusion strategy. EPO = erythropoietin, Hb = hemoglobin, IV = intravenous, OP = operation, POD = postoperative day, Preop = preoperative, TXA = tranexamic acid, wk = week.

An 11-year-old boy presented with a several-month history of left knee pain. After osteosarcoma was confirmed from an incisional biopsy specimen, we planned a limb salvage operation with tumor prosthesis. Although limb salvage operations with tumor prostheses usually require ABTs, the patient and his family did not want ABTs due to their religious beliefs, despite the patient's anemic conditions caused by neoadjuvant chemotherapy. Two weeks before surgery, the boy's Hb level was 7.5 g/dL. Following the previously described protocol, we administered rHuEPO and iron supplements, which increased his Hb levels to 11.0 g/dL by preoperative day 2. Surgery involved amputation or limb salvage operation with biologic reconstruction or tumor prosthesis (Fig. 1). After hypotension was induced, 1 g of tranexamic acid was diluted to 20 mL and continuously administered (4 mL/h) during the operation. The total operative time was 4.5 hours, with approximately 450 cm^3^ of intraoperative blood loss; a postoperative total hemovac drain check revealed a total collected volume of 550 cm^3^. By postoperative day 3, the patient's Hb dropped to its lowest level (6.6 g/dL). However, his laboratory findings gradually improved while following our protocol, with no anemia symptoms such as dyspnea or tachycardia (Fig. 1). The patient was maintained on the protocol for 2 weeks postoperatively until his Hb level recovered to 11.2 g/dL.

## 5 Discussion

Perioperative anemia should be addressed if surgical bleeding is expected to be significant.^[18]^ The rationale for anemia correction is that improving the oxygen-carrying capacity of the blood helps prevent clinical symptoms secondary to tissue hypoxia, including fatigue, shortness of breath, chest pain, weakness, loss of appetite, and headache.^[19]^ Although ABT is the most common method used to increase Hb levels, it is associated with adverse effects in patients with EBSs. Casper et al^[15]^ reported that perioperative ABTs were significantly prognostic of reduced survival in patients with EBSs. Similarly, Rosenberg et al^[13]^ reported that survival rates decrease as the amount of transfused blood increases. However, studies showing the impact of restrictive ABT strategies on postoperative EBS outcomes are limited. The present study demonstrated that restrictive blood management is a safe and effective way to reduce ABTs, without affecting clinical outcomes, in patients with EBSs.

There are several hypotheses for the association between perioperative ABTs and decreased survival rates in patients with cancer. These include leukocyte-mediated immunosuppression in allogeneic blood,^[20,21]^ transfusion-induced reduction in natural killer cell activity and interleukin-2 levels,^[22]^ and the infusion of incompatible major histocompatibility complex antigens.^[23]^ However, the increases in distant metastasis rates and decreases in survival rates may not be solely related to these factors. Considering the aggressiveness of cancer, we believe that the immunosuppressive effects of perioperative ABTs are associated with decreased survival. Furthermore, the increased rates of infection after joint arthroplasty in healthy patients receiving perioperative ABTs are well documented.^[24]^ Infections occurring around the tumor substitute, after limb salvage surgery, are additional critical factors that more significantly impact outcomes in osteosarcoma patients and those more susceptible to surgical site infections than in those undergoing general joint arthroplasty. Chesi et al^[14]^ showed high mortality rates in patients with osteosarcomas who received perioperative ABTs compared with other studies of soft tissue extremity sarcomas. This may have been because the patients with osteosarcoma were more prone to requiring joint arthroplasty, which may have led to complications including infection. Similarly, in the present study, postoperative surgical site infections only occurred in patients who received ABT during joint arthroplasties, irrespective of the transfusion strategy.

Our protocol involved several methods of anemia correction to reduce the number of ABTs. First, the use of rHuEPO is considered to enhance hematopoietic function in patients with anemia caused by chemotherapy, radiotherapy, or bone marrow tumor invasion.^[25]^ However, recent studies have shown minimal efficacy for rHuEPO, as well as the development of complications that may induce tumor growth^[26]^; therefore, we carefully restricted rHuEPO use to patients who underwent chemotherapy. Second, high-dose intravenous iron administration can improve preoperative Hb levels. Yoo et al^[27]^ reported that the use of intravenous iron, immediately before or after surgery, is effective for reducing postoperative hemorrhage and facilitates the early recovery of Hb levels. Third, as the National Health and Medical Research Council has suggested, intraoperative monitoring to maintain systolic blood pressure in the 80 to 100-mmHg range reduces intraoperative bleeding; further, maintaining normothermia helps prevent reversible platelet dysfunction caused by hypothermia.^[28]^ Thus, we applied induced hypotensive anesthesia and kept the patients warm to preserve normothermia. Fourth, tranexamic acid, which is well known and used worldwide to prevent bleeding without severe complications, was administered.^[29]^ Fifth, before wound closure and after tourniquet deflation, we performed meticulous hemostasis. We suggest that the present protocol is an effective strategy for patients with cancer to help prevent anemia and postoperative blood loss.

This study has several limitations. First, this study was conducted retrospectively; a randomized prospective study is needed to confirm the effects of restrictive ABTs. Second, the number of included patients was too small to generalize the results of this study. A multi-center study is required with a greater number of patients.

In conclusion, this study shows that a restrictive ABT strategy can be safely performed for wide-excision or limb-salvage operations in patients with EBSs. Therefore, surgeons should be aware of this approach and carefully consider its use according to the intraoperative status and specific characteristics of each patient.

## Author contributions

**Conceptualization:** Woo Young Jang.

**Data curation:** Seok Ha Hong, Woo Young Jang.

**Formal analysis:** Seok Ha Hong, Woo Young Jang.

**Funding acquisition:** Woo Young Jang.

**Writing – original draft:** Jong Hoon Park, Woo Young Jang.

**Writing – review & editing:** Jong Hoon Park, Woo Young Jang.

Woo Young Jang orcid: 0000-0003-1775-7715.
